# Diabetes & coronary heart disease: Current perspectives

**Published:** 2010-11

**Authors:** Mohammed K. Ali, K.M. Venkat Narayan, Nikhil Tandon

**Affiliations:** Hubert Department of Global Health, Rollins School of Public Health, Department of Medicine, School of Medicine, Emory University, Atlanta, USA; * *Department of Endocrinology & Metabolism, All India Institute of Medical Sciences, New Delhi, India*

**Keywords:** Coronary heart disease, diabetes, glycaemic control, hypertension, lipid, risk factors

## Abstract

Coronary heart disease (CHD) is currently the leading cause of death worldwide and together with diabetes, poses a serious health threat, particularly in the Indian Asian population. Risk factor management has evolved considerably with the continued emergence of new and thought-provoking evidence. The stream of laboratory- and population-based research findings as well as unresolved controversies may pose dilemmas and conflicting impulses in most clinicians, and even in our more well-informed patients. As results of the most recent clinical trials on glycaemic control for macrovascular risk reduction are woven into concrete clinical practice guidelines, this paper seeks to sort through unwieldy evidence, keeping these findings in perspective, to deliver a clearer message for the context of South Asia and cardio-metabolic risk management.

## Introduction

Cardiovascular diseases (CVD), comprising coronary heart (CHD) and cerebro-vascular diseases, are currently the leading cause of death globally, accounting for 21.9 per cent of total deaths, and are projected to increase to 26.3 per cent by 2030^1^. The factors that coalesce to increase the risk of developing atherosclerotic CHD were demonstrated in Framingham in the mid-20^th^ century^2^ and have subsequently been shown to be pervasive across ethnicities and regions of the world^3^. These are not new risks, but the ubiquity of smoking, dyslipidaemia, obesity, diabetes, and hypertension has been gradually escalating^4^, and is thought to be the driving influence behind the epidemic of heart disease faced today.

Of the risk factors, diabetes, and its predominant form, type 2 diabetes mellitus (T2DM), has a distinctive association with CHD. Those with diabetes have two- to four-fold higher risk of developing coronary disease than people without diabetes^5^, and CVD accounts for an overwhelming 65-75 per cent of deaths in people with diabetes^6^,^7^. More significantly however, the age- and sex-adjusted mortality risk in diabetic patients without pre-existing coronary artery disease was found to be *equal* to that of non-diabetic individuals with prior myocardial infarction (MI)^8^. These remarkable findings regarding higher risk of mortality^9^–^11^ have led to suspicion that common precursors predispose to diabetes and CHD^12^,^13^, with subsequent implications that insulin resistance, visceral adiposity, and excess inflammation^14^–^16^ underlie the pathophysiology of thrombogenesis. In addition, a complex mix of mechanistic processes such as oxidative stress, enhanced atherogenecity of cholesterol particles, abnormal vascular reactivity, augmented haemostatic activation, and renal dysfunction have been proposed as features characteristic of T2DM that may confer excess risk of CHD^17^.

People of Indian Asian descent make up over a fifth of the world’s population, combining inhabitants of the subcontinent and the Indian diaspora living elsewhere. The so-called “Asian Indian Phenotype” refers to an amalgamation of clinical (larger waist-to-hip and waist-to-height ratios signalling excess visceral adiposity), biochemical (insulin resistance, lower adiponectin, and higher C-reactive protein levels) and metabolic abnormalities [raised triglycerides, low high-density lipoprotein (HDL) cholesterol] that are more prevalent in individuals of South Asian origin and predispose this group to developing diabetes and premature CHD^18^–^20^. It is expected that individuals of Indian Asian ethnicity will account for between 40-60 per cent of global CVD burden within the next 10-15 years^21^. The astonishingly higher risk in this particular ethnic group has been attributed to underlying genetic susceptibility^22^,^23^ unmasked by environmental factors (permeation of contemporary lifestyle practices)^24^ or intrauterine programming which predisposes to asymmetric energy metabolism and rapid, excess accumulation of visceral body fat in adult life^25^–^27^.

In terms of absolute numbers of individuals with diabetes, India, Pakistan and Bangladesh make up three of the top ten countries globally^28^ and together, the region with the highest number of diabetes-related deaths currently^29^. India alone is estimated to have 50.8 million inhabitants with diabetes, the most of any country worldwide^29^. Propelled by socio-economic transformation, population ageing, burgeoning levels of overweight^30^ and proliferation of individuals and children with pre-diabetes (impaired glucose regulation)^31^, increase in T2DM^29^,^32^,^33^ and CHD^4^ will result in even greater future burdens.

The proportion of coronary disease patients with diabetes varies across countries, but approximately one-fifth of clinical trial (18%)^34^ and registry patients (15.1-21.4%)^35^ are documented as known diabetes patients. India stands out as an anomaly with 30.4 per cent^36^ and 39.1 per cent^35^ of CHD patients reporting known diabetes in national and international prospective registries. These proportions may be deemed the result of high background prevalence of glucose abnormalities in India. However, given that South Asians have higher prevalence of cardiovascular risk factors^37^,^38^, higher prevalence of T2DM, and earlier onset of CHD despite a normal body mass index (BMI) by international standards^18^,^37^,^39^,^40^, the premise that this population is more susceptible to diabetes and CVD^19^,^20^, and that these conditions are interlinked, is plausible.

Though previously CHD and T2DM were considered mainly diseases of affluence, reversal of socio-economic gradient in these diseases is starting as lower socio-economic groups in South Asia are exhibiting ever-increasing risk^41^,^42^. In addition, characteristic disparities (rural-urban split, public-private health care and low awareness) that are pervasive across the region, combined with chronicity and asymptomatic nature (silent killer) of non-communicable risk factors and diseases, perpetuate delays in diagnosis, inertia to seek care, and effective self-management of risks.

## Risk factor control in cardiovascular disease reduction

Broadly speaking, established CVD risk factors most often do not occur in isolation, and addition of associated morbidities results in multiplicative, rather than additive, amplification of risk^10^. Once any individual factor is identified, systematic, comprehensive, and regular assessments should be undertaken to identify the development of co-existing risks or target organ complications, and treatment plus monitoring should be diligently instituted^43^. Driven by physician eagerness, haste, and to some extent, pharmaceutical sector interests^44^,^45^ and persuasion^46^, there has been strong emphasis on medication usage in managing dyslipidaemia, hypertension, and diabetes. This has detracted somewhat from the significant benefits that can be gleaned from alteration in lifestyle (nutrition^47^, weight, physical activity and tobacco use^48^) that occurs upstream of metabolic disturbances.

There is robust evidence that lifestyle modification (regular, moderate physical activity and healthy dietary habits) has a sustained effect on reducing incidence of diabetes^49^–^51^, and helps reduce the occurrence and mortality of CVD events in people with and without established CHD^52^. Iestra and colleagues^53^ have shown relative risk of mortality is reduced in the general population that stop smoking (up to 50% reduction), engage in moderate physical activity (20-30%), and adopt a combination of healthy dietary habits (limited intake of saturated fats, regular fish consumption, sufficient fruit and vegetable intake, and limited salt consumption - together, 15-40% reduction).

Randomized clinical trials evaluating individual risk factor control with pharmaceutical agents in patients with diabetes have also demonstrated reduction in surrogate markers, which translated into lower incidence of cardiovascular events and mortality. These findings have been utilized to institute clinical practice guidelines and standards of care based on strength of evidence and cost-effectiveness of interventions Table I.

**Table I:** Evidence-based cardiovascular risk management targets in diabetes

	Target risk factor	Class of recommendation & level of evidence	Recommended targets	
			ESC	ADA
Glycaemia	Glycosylated haemoglobin	Normoglycaemia reduces risk of microvascular complications (Level A, Class I)	< 6.5 %	≤ 7.0 %; individualize based on patient profile
	Fasting plasma glucose	Metformin = first line for overweight T2DM (Level C, Class IIb)	< 6.0 mmol (108 mg/dl)	3.9–7.2 mmol/l (70–130 mg/dl)
	Post-prandial glucose	Early stepwise increases in therapy improves morbidity & mortality (Level B, Class IIa)	T2DM < 7.5 mmol (135mg/dl); T1DM 7.5-9.0 mmol (135-160 mg/dl)	10.0 mmol/l (180 mg/dl)
Lipids	Total cholesterol	Measure fasting lipid profile annually to every 2 yr (Level B/C, Class IIb) depending on risk	< 4.5 mmol (175 mg/dl); If TC>3.5 mmol, aim for 30-40% LDL↓	
	LDL-cholesterol	Add statin to lifestyle therapy where overt CVD or no CVD, but >40 yr of age with ≥1 risk factor (Level A, Class I); If LDL targets not met despite maximal drug dose, aim for 30-40% reduction from baseline (Level A)	≤ 1.8 mmol (70 mg/dl)	<2.6 mmol/l (100 mg/dl); <1.8 mmol/l (70 mg/dl) if overt CVD
	HDL-cholesterol	↑HDL & ↓Triglyc. desirable (Level C, Class IIb)	Men: >1.0 mmol (40 mg/dl) Women: >1.2 mmol (46 mg/dl)	Men: >1.0 mmol (40 mg/dl) Women: >1.3 mmol (50 mg/dl)
	Triglycerides	Combining statins with other lipid-altering agents may be considered (Level C, Class III)	<1.7 mmol (150 mg/dl)	<1.7 mmol (150 mg/dl)
BP	BP control	BP targets & monitoring at every visit (level B-C)	<130/80 mmHg	<130/80 mmHg
	(& use of RAS-modifying agent)	Pharmacologic therapy if >140/90 (Level A, Class I); multiple therapies often required for achieving targets (Level B)	<125/75 mmHg (if renal impairment)	
Medication	Anti-platelet agents	Aspirin use in patients with history of CVD (Level A, Class I); if male >50 yr or female >60 yr with 1 additional risk factor (Level C)	ASA 75 mg/day	ASA 75-162 mg/day
		Clopidogrel (Level C, Class IIa) if severe CVD; combine with aspirin in 1^st^ yr after MI (Level B)		
	ACE-inhibitor use	Where additional risk factors exist: To delay renal complications (Class I, Level A)		
		To reduce CV events (Level B)		
	Vaccinations	Annual influenza – Level C One lifetime pneumococcal vaccine (for >65 yr, renal disease/post-transplant patients) – Level C		
Lifestyle	Smoking	Advise cessation (Level A) Utilize counselling & therapies (Level B)	Cessation	Cessation
	Regular physical activity	Level A, Class I	30-45 min/day	150 min/wk of moderate intensity aerobic activity (+/- resistance training 3 times/wk)
	Weight control	Either low-carbohydrate or low-fat calorierestricted diet may be effective (up to 1 yr)-Level of evidence A	BMI<25.0 kg/m^2^* 10 % weight reduction (if already overweight)	Especially in overweight or obese individuals with insulin resistance
Assimilated from Task Force on DM & CVD (ESC, European Society of Cardiology and European Association for Study of Diabetes, 2007)83 & American Diabetes Association (ADA) Standards of Medical Care in Diabetes - 2010^84^				

Values for glucose & lipids presented as mmol/l (mg/dl)

* Lower BMI targets applicable to Indian Asian and Chinese Asian populations; RAS, renin-angiotensin system; ACE-inhibitor, angiotensin converting enzyme inhibitor; ASA, acetylsalicylic acid (aspirin)

Level of evidence A: Data derived from multiple, well-conducted, adequately powered randomized clinical trials or meta-analyses Level of evidence B: Data derived from a single randomized clinical trial or large non-randomized studies (cohort, registries, meta-analysis of cohort studies) Level of evidence C: Consensus of opinion of experts and/or small studies, retrospective or observational studies (+/- methodological flaws, biases)

Classes of Recommendation

Class I: Evidence &/or general agreement that a given diagnostic procedure/treatment is beneficial, useful, and effective Class II: Conflicting evidence and/or a divergence of opinion about the usefulness/efficacy of the treatment or procedure Class IIa: Weight of evidence/opinion is in favour of usefulness/efficacy Class IIb: Usefulness/efficacy is less well established by evidence/opinion Class III: Evidence or general agreement that the treatment or procedure is not useful/effective and in some cases may be harmful

Dyslipidaemia is a significant predictor of CVD events and mortality in diabetes patients^55^,^56^. Attentive management of low-density lipoprotein (LDL-), high-density lipoprotein (HDL-) and total cholesterol, but also triglyceride subfractions, is vital. HMG-CoA reductase inhibitors (statins) in particular, have indisputable proven efficacy, demonstrating 27-40 per cent reductions in LDL-cholesterol in all placebo-controlled trials (Table II), and subsequent decreases in occurrence of cardiovascular events and mortality by 25 to 42 per cent^57^,^58^ in persons with and without diabetes or previous acute coronary syndrome (ACS). This benefit extends to those with already controlled LDL-cholesterol fractions^59^.

**Table II:** Intermediate and end point effect size estimates for drug-based interventions

Trial (DM sample size)	Patient characteristics	Intervention	Intermediate outcome	CVD risk reduction
***LDL lowering***				
HPS-DM (5,963)^57^	DM without CHD	Simvastatin 40 mg	31%↓ in LDL	22-26%↓ composite end points
CARDS (2,838)^58^	T2DMwith≥ 1 RF	Atorvastatin 10 mg	40%↓ in LDL	37%↓ in events, 27%↓ CV mortality
4S-DM (483, IFG=678)^85^	DM subgroup & IFG	Simvastatin 20-40 mg	36%↓ in LDL	42%↓ in CHD events, 28%↓ CV mortality
CARE-DM(586)^86^	DM subgroup	Pravastatin 40 mg	27% ↓ in LDL	25%↓ in events, 32%↓ revascularization
***HDL & Triglycerides***				
VA-HIT (2,531)^60^	CHD with normal LDL	Gemfibrozil	6%↑HDL; 31%↓Tg	24%↓ in CV events, 32%↓ composite
FIELD (9,795)^62^	T2DM, age 50-75, ↑chol	Fenofibrate 200 mg	7%↓TC; 22%↓Tg	24%↓ in non-fatal MI, 11%↓ CV events
***BP Control***				
HDS-UKPDS(1,148)^69^	DM subjects	Aggressive BP Rx	↓BP (144/82)	32%↓ in DM-related deaths
HOT-DM (1,501)^70^	DM subjects	dBP≤80 vs ≤90mmHg	↓dBP 20.3-24.3	51%↓ in CV end points
ADVANCE-BP^71^	T2DMwith≥1 RF/TOD	Add ACEi / Indap.	↓sBP 5.6, ↓dBP2.2	8%↓ in CVD events, 18%↓ CV mortality
ABCD (950)^87^	T2DM	dBP≤75 vs ≤90mmHg	128/75 vs. 137/81	BP↓ stabilized Cr Cl & ↓ micro vase. TOD
ABCD (470)^88^	T2DM with HTN	ACEi vs CCB	Similar BP ↓	ACEi 9.5 times ↓ composite outcomes
Syst-EUR (492)^72^	Systolic HTN & DM	CCB ± ACE/Thi	↓sBP8.6, ↓dBP3.9	63%↓ in CV events
ALLHAT-DM(13,101)^74^	DM, IFG (1,399)	ACEi / CCB / thiazide	↓sBP: Thi>CCB>ACE	No difference in CV events; thiaz ↑ FBG
BPLTTC-DM (33,395)^89^	DM patients from BP trials	meta-analysis:27 trials	Similar efficacy of drug regimens	Comparable ↓ in CV events in DM/non; limited evidence for lower BP target in DM
***HAS (ACE/ARB)modifier***				
RENAAL(1,513)^78^	T2DM with nephropathy	Losartan (ARB)	↓BP	16-28%↓ (or 2 yr) delay in dialysis/Tx
HOPE (3,577)^80^	DM with ≥ 1 RF	Ramipril (ACEi)	Adjusted for Δ in BP	25%↓ in composite endpoint
ABCD-2 (129)^77^	T2DM & normotensive	Valsartan (ARB)	118/75 vs. 124/80	↓ in urinary albumin & possibly CVD^91^
***Anti-platelet therapy***				
ATC (4,500)^91^	Meta-analysis (high-risk)	Aspirin		18%↓ incidence of CV events
HOT-DM (1,501)^71^	DM subjects	Aspirin		15%↓ in events, 36%↓ in mortality
CAPRIE (3,866)^92^,^93^	DM subjects	Clopidogrel vs ASA		9-12% less events in Clopidogrel arm

^*^Composite outcomes most often include: non-fatal MI, stroke, revascularization &/or cardiovascular mortality

CV, cardiovascular; RF, risk factor; HTN, hypertension; Cr Cl, creatinine clearance; TOD, target organ damage; Tx, transplant; Δ, change; ASA, acetylsalicylic acid (aspirin); ACEi, angiotensin converting enzyme inhibitor; ARB, angiotensin receptor blocker; Indap., indapamide; ↓, decrease; ↑, increase; sBP, systolic BP; dBP, diastolic BP HPS, Heart Protection Study; CARDS, Collaborative Atorvastatin Diabetes Study; 4S, Scandinavian Simvastatin Survival Study; CARE, Cholesterol and Recurrent Events study; VA-HIT, Veterans Affairs High-Density Lipoprotein Intervention Trial; FIELD, Fenofibrate Intervention and Event Lowering in Diabetes; HDS-UKPDS, Hypertension in Diabetes Study - United Kingdom Prospective Diabetes Study; HOT, Hypertension Optimal Treatment study; ADVANCE-BP, Action in Diabetes and Vascular Disease: Preterax and Diamicron Modified Release Controlled Evaluation trial, BP-lowering arm; ABCD, Appropriate Blood Pressure Control in Diabetes; SystEUR, Systolic Hypertension in Europe trial; BPLTTC, Blood Pressure Lowering Treatment Trialists’ Collaboration; ALLHAT, Antihypertensive and Lipid-Lowering Treatment to Prevent Heart Attack Trial; HOPE, Heart Outcomes Prevention Evaluation; RENAAL, Reduction of Endpoints in NIDDM with the Angiotensin II Antagonist Losartan; ABCD2-Valsartan, Appropriate Blood Pressure Control In Hypertensive and Normotensive Type 2 DM - Valsartan; ATC, Antiplatelet Trialists Collaboration; CAPRIE, Clopidogrel versus Aspirin in Patients at Risk of Ischaemic Events

There is ambiguity concerning the role of gemfibrozil, nicotinic acid, and fibrates in CVD risk reduction. At least modest improvements in HDL-cholesterol and triglycerides have been postulated, but significant concrete translation into lower composites of CVD have not been exhibited in studies^60^–^63^. Since the evidence in favour of lowering LDL is so overwhelming^59^ and similar findings are awaited for triglyceride management and elevating HDL^64^,^65^, the primary emphasis of lipid management tends to focus on LDL. Dietary modification^66^ and addition of statins are, therefore, recommended as first-line management guidelines for lipid control in diagnosed diabetes patients or those with confirmed CVD.

Hypertension co-exists in a significant proportion of people with diabetes^67^. Lowering blood pressure (BP) produces dramatic benefits in these subjects and BP targets have been modified specifically to avert disabling and fatal complications in the form of nephropathy, retinopathy, and vascular events^68^. Several large randomized trials, sub-studies^69^–^74^, and meta-analyses^75^,^76^ which include patients with diabetes have shown benefit in reducing non-fatal myocardial infarction, chronic kidney disease^77^,^78^, and remarkable reductions in cardiovascular (51%)^70^ and all-cause mortality. The use of renin-angiotensin system (RAS) modifying agents [angiotensin-converting enzyme inhibitors (ACEi) or angiotensin-II receptor blockers (ARB)] provide ancillary benefits in forestalling renal complications^78^ on top of BP control, and have additionally demonstrated lower composite CVD outcomes in numerous randomized trials, even after adjustment for changes in BP^79^–^81^. These cardio- and reno-protective effects (anti-atherosclerotic, reducing arterial stiffness, and improving endothelial function)^73^,^82^ are motivating more extensive application of RAS-modifier agents in patients with diabetes.

Previous evidence has also demonstrated the efficacy of low-dose daily aspirin use in preventing CVD events, especially as secondary prevention in those that have already suffered previous events^94^,^95^. However, a recent large meta-analysis^96^ cautions that ubiquitous use of low-dose aspirin for primary prevention may only be justified where net benefits of preventing coronary events in high-risk patients outweigh the increased risk of gastrointestinal and extra-cranial bleeds. As such, this study showed no significant effects on preventing first onset of stroke. The addition of clopidogrel is currently also recommended only for prevention of recurrent events^92^,^93^.

## Glycaemic Control in Cardiovascular Risk Reduction: An Actively Evolving Paradigm

In patients with diabetes, where excess CVD risk has already been demonstrated, the relationship between glycaemia itself and CVD should not, theoretically, be in doubt. Even studies in non-diabetic subjects^97^,^98^, including a meta-regression analysis combining data from >95000 participants^99^, have shown an association between fasting blood glucose and CVD. Another meta-analysis of prospective cohort studies^100^ examined glycosylated haemoglobin (HbA_1_c a more stable, accurate, less error-prone measure of long-term glycaemic levels) and CVD in persons with diabetes and found 18 per cent (pooled RR 1.18; 95% CI 1.10 to 1.26) and 15 per cent (pooled RR 1.15; 95% CI 0.92 to 1.43) greater relative risk per 1 per cent increase in HbA_1_c in T2DM and T1DM, respectively. However, the converse of this association, whether reducing glucose levels to near-normal targets results in lower CVD events, is still a controversial topic.

Despite impressive reduction in microvascular complications^69^,^101^–^103^ and retrospective cohort data showing lower risk of strokes (21%) and MI (23%)^104^ with lower levels of glycaemia, the early prospective trial data evaluating macrovascular outcomes classically provided equivocal results [*e.g*. 16% (*P*=0.052) non-significant reduction in MI in United Kingdom Prospective Diabetes Study (UKPDS)], citing reasons of inadequate power, follow-up^101^,^102^ or design deficiencies. More recent multi-centre trials sought to conclusively evaluate the influence of achieving lower therapeutic targets for glycaemic control on the incidence of CVD endpoints. Since a variety of pharmacological and non-pharmacological treatments are established, cost-effective, and safe interventions for glycaemic control^106^,^107^, the more contemporary theme of what level of glycaemia to achieve holds topical interest, requiring more in-depth discussion.

Three large prospective randomized trials attempted to definitively address the glycaemia and CVD debate. The Action to Control Cardiovascular Risk in Diabetes (ACCORD)^108^, Action in Diabetes and Vascular Disease: Preterax and Diamicron Modified Release Controlled Evaluation (ADVANCE)^109^, and Veterans Affairs Diabetes Trial (VADT)^110^ studies randomized 10251, 11140, and 1791 T2DM patients, respectively, with co-existing risk factors and/or history of diabetic complications (including previous CVD events) into intensive (aiming for bold “near-normal” glycaemic targets) or conventional therapy groups, using different treatment regimens. After a 1.1 per cent relative difference in median HbA_1_c between the groups (6.4 vs. 7.5%) and 3.5 years of follow-up, ACCORD was prematurely discontinued due to 54 excess deaths in the intensive therapy arm. The ADVANCE trial achieved a 0.8 per cent lower median HbA_1_c (6.5%) in the intensive therapy arm compared to the standard group over a 5 year follow-up period and demonstrated a 10 per cent reduction in composite of major micro- and macro-vascular events (HR 0.90, 95% CI 0.82 to 0.98; *P*=0.01), which did not remain significant after adjustment for reduction in nephropathy (21% reduction in intensive therapy group; HR 0.79, 95% CI 0.66 to 0.93; *P*=0.006). From a baseline median HbA_1_c level of 9.4 per cent, the VADT achieved A1c levels of 6.9 and 8.4 per cent in the intensive and standard groups, respectively^109^. However, there was no significant between-group difference in the composite primary outcome (HR 0.88; 95% CI, 0.75 to1.05; *P*=0.14), nor in the number of new, or progression of, microvascular complications. Across these three studies, the intensive therapy arms all reported higher incidence of hypoglycaemia requiring medical assistance and weight gain among participants.

Despite seemingly negative results, there are several points from these study results that should be kept in perspective, especially as outcomes of ongoing trials will continue to emerge at regular intervals in the future (Table III)^111^. Firstly, diabetes is still the leading cause of adult-onset blindness, end-stage renal disease, and non-traumatic lower-extremity amputations worldwide^32^,^112^,^113^, and glycaemic control overwhelmingly reduces these microvascular complications^69^,^101^,^102^. Therefore, blood glucose management remains a vital component of preventing disabling and fatal target organ damage in both T1DM and T2DM. Secondly, optimal glycaemic targets have been chosen based on this evidence from microvascular risk reduction and should at least be deemed appropriate considering the increased risks of hypertension, dyslipidaemia, and hyperhomocysteinaemia - themselves strong risks for CVD - which are associated with renal insufficiency. However, in the broader context of CVD prevention and considering the severity of chronic kidney disease, these targets may need to be customized according to individual risk^114^.

Other important considerations include the fact that participants in these large trials were high-risk patients with poor baseline control, high pre-existing use of insulin (among 35-50% of subjects), and a third (32-40%) already had pre-existing heart disease. Indeed, the average duration of diabetes (8.5-11 yr) must also weigh in as a factor, which motivates the assertion that either earlier^17^, or more sustained intervention is required to reduce the risk of prolonged metabolic disturbance. This was confirmed in the 17 yr follow-up of the Diabetes Control and Complications Trial (DCCT)^115^ where the intensively treated type 1 diabetes patients had 42 and 57 per cent lower risk of CVD events and death from CVD, respectively, despite no difference found at earlier follow-up. The UKPDS ten-year follow-up^116^ also demonstrated delayed beneficial effects of early initiation of glycaemic control on macrovascular outcomes, a “metabolic memory” of sorts.

**Table III:** Summary of trials assessing glycaemic control targets & alternative therapies

Trial (sample size)	Patient characteristics	Intervention (F/up)	Results	CVD risk changes
*Evaluating glycaemic target*				
VADT (1,791)^110^	DM & > 40 yr of age	Sequential therapy intensification vs. standard care PLUS education & management of RFs to both groups (6.25 yr)	Baseline: mean age 60, 40% previous events, most ≥ 1 RF Mean HbA1c (6.9 vs. 8.4%)	No significant difference in CVS events (235 vs 264, NS), microvascular complications or death between intensive & standard groups; Baseline coronary calcium was strongest predictor of CVD outcomes
ACCORD (10,251)^108^	T2DM with previous CVD or ≥ 2 RF or albuminuria, atherosclerosis or LVH	Intensive glycaemic control (HbA_1_c ≤ 6) vs. standard therapy (A_1_c 7.0-7.9) -(discontinued after 3.5 yr)	Baseline: mean age 62, 35% prev. CVD; Median HbA_1_c Δ 1.1% (6.4 vs. 7.5%)	20%↑ in death of any cause (including CVD / CHF / fatal procedures)
ADVANCE (11,140)^109^	T2DM & history of complications or co-existing RF (200 centers)	Intensive glycaemic control (HbA1c ≤ 6.5) with gliclazide MR vs. conventional therapy (median 5 yr)	Baseline: mean age 66, 32% prev.CVD & 10% prev. microvascular TOD; Median HbA1c Δ 0.8% (6.5 vs. 7.3%)	10%↓ composite of micro- & macro-vascular outcomes (NS after adjustment for 21%↓ in nephropathy)
Ray & colleagues Meta-analysis (33,040)^117^	5 trials – random effects meta-analysis	Intensive glycaemic control versus standard control; 163,000 person yr of follow up	Mean 0.9% lower A1c in intensive arms	17% ↓ non-fatal MI 15% ↓ in CHD events No difference in stroke (HR 0.93; 0.81-1.06) and all-cause mortality (HR 1.02; 0.87-1.19)
CONTROL Meta-analysis (27,049)^118^	4 prospective trials including T2DM pts; mean age=62yr; median duration of DM=9 yr;	Intensive versus less-intensive glucose control over 4.4 yr	Mean 0.88% lower A1c in intensive arms	9% ↓ in CVD events (as high as 16% benefit in those without pre-existing macrovascular disease) No difference in all-cause mortality (HR 1.04) and CVD-mortality (HR 1.10)
*Trials evaluating alternative therapies*				
ORIGIN (~10,000)	IFG, IGT, DM with CVD risk factors	Omega fatty-acid consumption		Do unsaturated fats have cardio-protective properties in patients with dysglycaemia?
LookAHEAD (~5,000)	T2DM aged 45-74 with BMI ≥ 25 kg/m^2^	4 yr intensive weight-loss (11.5 yr planned f/up)	Only 10.1% achieved targets – sociodemographic & compliance factors	Powered for 90% probability of detecting 18% Δ in major CVD events
ASCEND (~10,000)^127^	DM	Omega-3 fatty acids & Aspirin 100 mg/day (2×2 design)		Are Omega-3 FA & Aspirin of benefit independently & together in DM patients?
SEARCH (9,000)	Youths (<20 yr) with DM (T1DM)	Cohort		Document prevalence of T1DM & follow service utilization, quality of care & development of complications
HPS2-THRIVE (20,000)^128^	Previous CVD (China, UK, Scandinavia); DM sub-population 7,500	Niacin & MK-0524A		Does increasing HDL lower CVD event rate?

*Source*: Refs 111,114,129, and individual trail information websites: (*http://www.searchfordiabetes.org/; http://www.lookaheadtrial.org/; http://www.ctsu.ox.ac.uk/ascend/; http://clinicaltrials.gov/ct2/show/NCT00069784; http://www.ctsu.ox.ac.uk/projects/hps2-thrive*)

DM, diabetes mellitus; T1DM, type 1 diabetes mellitus; T2DM, type 2 diabetes mellitus; IFG, impaired fasting glucose; IGT, impaired glucose tolerance; f/up, follow up; yr, year; Δ, change/difference; CHF, congestive heart failure; Omega-3 FA, omega-3 fatty acids; Gliclazide MR, modified-release preparation; UK, United Kingdom; RF, risk factor; TOD, target organ damage; NS, non-significant; prev, previous; VADT, Veterans Affairs Diabetes Trial; ACCORD, Action to Control Cardiovascular Risk in Diabetes study; ADVANCE, Action in Diabetes and Vascular Disease: Preterax and Diamicron Modified Release Controlled Evaluation trial; CONTROL, Collaborators on Trials of Lowering Glucose; ORIGIN, Outcome Reduction with Initial Glargine Intervention trial; LookAHEAD, Action for Health in Diabetes study; ASCEND, A Study of Cardiovascular Events iN Diabetes; SEARCH, SEARCH for Diabetes in Youth study; HPS2-THRIVE, Heart Protection Study 2 - Treatment of HDL to Reduce the Incidence of Vascular Events study

In addition to greater frequency of hypoglycaemia and weight gain in the intensive group participants, it has been postulated that serious adverse events and mortality may be attributable to more aggressive and rapid (*e.g*., ACCORD and VADT permitted any drug combination with rapid glucose-lowering) than measured (*e.g*., ADVANCE used sulphonylureas with gradual between-group differences in glycaemia) glucose-lowering; however, there are currently no data to support this assertion.

Recently published meta-analyses^117^,^118^ (Table III) have sought to examine the data in its entirety, pooling data and performing several pre-specified sub-analyses. The findings seem to conclude that intensive glucose lowering may have significant benefits in preventing coronary events, especially in those without pre-existing established atherosclerotic vascular disease; however, there is seemingly no mortality-reducing benefit from targeted glucose management. Based on the totality of evidence available, the American Diabetes Association, American College of Cardiology, and American Heart Association jointly issued recommendations^119^ to assuage uncertainty and confusion that emerged among clinicians and scientists following the release of these trial results. Broadly, current guidelines support customizing the intensity of glucose management depending on individual patient characteristics and co-morbidities.

A final and very convincing point is that glycaemia is not the sole consideration in CVD risk, but rather plays a role in the confluence of multi-factorial influences^120^. Therefore, exclusively concentrating on glucose control may be a key limitation of these focused randomized trials. A meta-analysis which presented a substantial 27 per cent greater risk for CVD between the highest (8.3-10.8 mmol/l) and lowest (3.8-5.9 mmol/l) post-challenge blood glucose levels, subsequently also revealed significant attenuation of risk (to 19%) when adjustment was made for co-existing CVD risk factors^121^. Progression of carotid atherosclerosis in diabetes patients showed analogous attenuation upon controlling for other CVD risks^122^. By aggressively managing all modifiable risk factors (blood pressure and lipid control)^57^,^58^,^123^ and implementing evidence-based guidelines^94^,^124^ vascular events and mortality can be reduced considerably^125^,^126^. Also, within these large trials of glycemic control, embedded trials were conducted to examine the effect of targeted, rigorous treatment of co-morbid risk factors on event rates; results from these trials are eagerly anticipated. A good example of comprehensive risk factor control is the Steno-II study investigated integrated, comprehensive, intensified risk factor control in a randomized fashion in Danish T2DM patients with microalbuminuria. They demonstrated declines in metabolic parameters (including HbA_1_c values) which translated into sizeable gains in prevention of CVD (53%) over 7.8 yr^125^ and lower mortality from cardiovascular events (59%) over 13.3 yr of follow-up^126^. The case for concerted multiple risk factor modification and drawing well-informed lessons Fig. from the literature is therefore compelling^16^,^67^,^114^,^125^.

**Fig.**
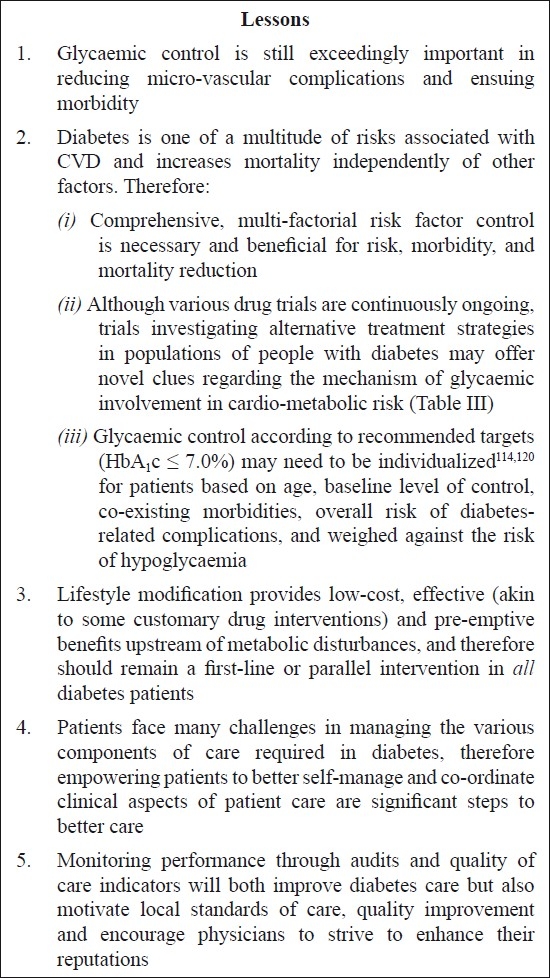
Themes and lessons emerging from recent trial evidence

Interpretation of findings is influenced by qualitative and quantitative heterogeneity across trials and publication biases noted in most meta-analyses. It should also be noted that some studies compare 10-year risk scores, others measure actual events and mortality, and the differences are mainly a function of duration of follow up. Other noteworthy dissimilarities in studies evaluated are in characteristics of patients enrolled, particularly demographic characteristics, socio-economic status, baseline level of control and risk factor duration prior to participation in the study, as well as enduring motivation of participants. There is also a crucial distinction between reporting relative reductions of biochemical parameters versus standard therapy as in some studies, and actually achieving recommended optimal targets in others.

## Conclusions

Glycaemic and CVD risk factors control can be challenging in any context, not least in the sub-continent. Evidence-based recommendations for diabetes care, mainly based on trials in Anglo-Caucasian populations, are available, but there is no indication of how well these guidelines are implemented in South Asia, nor any randomized trial evidence is available from this particular population group. The Delhi Diabetes Community (DEDICOM)^130^ and DiabCare Asia^131^ surveys suggest that quality of diabetes care is sub-optimal (participants reported low frequency of self-monitoring and poor glycaemic control (HbA_1_c >8%) amongst 42-50 per cent of diabetes patients, only 17.5 per cent were taking aspirin while lipid and BP targets were not met in almost half the subjects surveyed) in a region where dedicated, diligent follow up of diabetes patients should be a priority, given the amplified risks. Poor clinical practices such as these^132^ help explain the remarkable proportion (54%) that reported severe late-stage complications. Focused, context-specific research^133^ and careful analyses that integrate medication therapy and preventative lifestyle choices may pave the way for alignment of resources with needs, health systems development, and consequent reductions in morbidity and mortality.
